# Response Comment on “Validity and Reliability of the Iranian Version of the Short Form Social Well Being Scale in a General Urban Population”

**Published:** 2020-04

**Authors:** Zeinab SHAYEGHIAN, Parisa AMIRI, Golnaz VAHEDI-NOTASH, Mehrdad KARIMI, Fereidoun AZIZI

**Affiliations:** 1. Research Center for Social Determinants of Endocrine Health, Research Institute for Endocrine Sciences, Shahid Beheshti University of Medical Sciences, Tehran, Iran; 2. Endocrine Research Center, Research Institute for Endocrine Sciences, Shahid Beheshti University of Medical Sciences, Tehran, Iran

## Dear Editor-in-Chief

We would like to thank you for the opportunity to respond to the issue raised in the letter provided by Behghadami et al. and to clarify our reasons in relation to this concern. We would also like to thank the authors for their interest in our paper and for taking the time to provide their comments.

In their letter, the authors pointed out the necessity of measuring content validity of scales in psychometric studies. Content validity refers to the extent to which items in a questionnaire are representative of the theoretical construct(s) that are supposed to be assessed (1). Hence, in the developing phase of the questionnaire, the constructs of interest determine which items need to be considered (2). We agree that content validity is an important step in the validation process of questionnaires; however, this process is particularly crucial in developing of a new questionnaire (2, 3). In this regard a review article focused on the common methods using for validation of translated questionnaires in medical contents show that, of 47 studies, seven used a panel of experts to establish content validity and the Content Validity Index (CVI) of the target language version had been calculated only in two of them (4).

The Short Form Social Well Being Scale is not a new questionnaire and the theoretical structure, construct validity, and the social structural sources of the dimensions of this scale have been investigated in several previous studies (5–8). In addition, there are few studies which have used this scale in Iranian populations (9–11). Since, there is no report indicating the process of linguistic validation of the Iranian version of this scale, we aimed to provide scientific evidence for this process that can be applied in future researches. Hence, similar to other cross-cultural studies which have been conducted to assess linguistic validity of the questionnaire in other countries (6, 7, 9), its content validity was not assessed in our study.
